# The Quest for Optimal Fractionation Schedules in Stereotactic Radiotherapy

**DOI:** 10.7759/cureus.6777

**Published:** 2020-01-26

**Authors:** Yuta Shibamoto, Hiromitsu Iwata

**Affiliations:** 1 Radiology, Nagoya City University Graduate School of Medical Sciences, Nagoya, JPN; 2 Radiation Oncology, Nagoya City West Medical Center Hospital, Nagoya, JPN

**Keywords:** stereotactic radiotherapy, radiosurgery, fractionation, reoxygenation, redistribution

## Abstract

In stereotactic radiotherapy (SRT), treatment with a single high dose is usually feasible as it spares the surrounding normal tissues, especially for intracranial lesions. However, single-fraction treatment may not be ideal in terms of efficacy. Single-dose treatment is particularly inefficient for tumors with hypoxia. Generally, tumors with a size of over 1 cm in diameter are considered to contain hypoxic cells, but the radioresistance of hypoxic cells can be partly overcome by fractionation owing to the reoxygenation of hypoxic tumor cells between fractions. In this article, we discuss the radiobiological characteristics of fractionated stereotactic irradiation (STI). And, based on our observations, we recommend that a dose of 54-60 Gy in six-eight fractions delivered two or three times a week for lung and other tumors larger than 2 cm in diameter is the ideal method of SRT.

## Introduction and background

Radiation therapy has a history of over 120 years. During the initial years of radiotherapy in the late 1890s, a single or a few fractions of radiation were used to treat superficial cancers [1]. A few years later, fractionated treatment came to be recognized as more efficient in terms of the antitumor effect and normal tissue toxicities. And after several decades, a dose that constituted 1.8 to 2 Gy of daily fractions administered five times a week, up to a total dose of 50-66 Gy, came to be established as the standard fractionation schedule in conventional radiotherapy. When the fractional dose is escalated, antitumor effects increase; but probabilities of complications in normal tissues, especially late-responding normal tissues, are considered to further increase with this escalation and outweigh the increase in antitumor effects. Hence, even in modern radiotherapy, 1.8- to 2-Gy daily fractions still remain the standard when moderate or large volumes of normal tissues are included in the radiation field using conventional radiation techniques.

However, advances in radiation techniques have brought about marked changes in fractionation schedules. With the technique of stereotactic irradiation (STI), radiation doses can be focused on the target while minimizing irradiation of the surrounding normal tissues. Therefore, the concept of conventional fractionation and the issues associated with it do not apply to STI. A relatively small lesion can be treated with a single fraction of 20-30 Gy with no apparent adverse effects with STI. Hence, single-fraction STI has often been employed especially in the treatment of intracranial lesions. Such a short-course treatment is beneficial for patients in palliative settings. However, the efficacy of single-fraction treatment for all tumors has not been well-established. Recent clinical evidence suggests that fractionated STI is more efficient than single-fraction stereotactic radiosurgery (SRS), especially for large tumors. In stereotactic body radiation therapy (SBRT) for lung tumors, four-fraction treatment has been empirically employed the most often; however, there is no radiobiological evidence to suggest that a particular fractionation schedule is the most ideal one. In this article, we aimed to discuss the optimal fractionation schedules in STI.

## Review

Factors influencing the efficacy of stereotactic irradiation

1. Tumor Hypoxia

**Figure 1:**
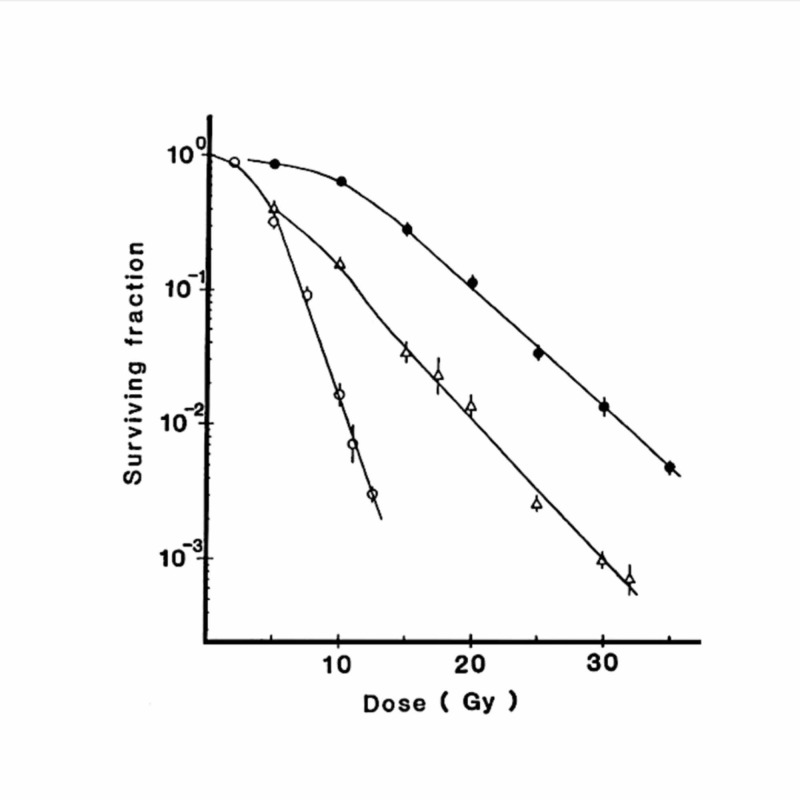
Dose-survival curves for EMT6 single cells irradiated under aerobic conditions (○) and hypoxic conditions (●) and EMT6 tumor cells irradiated in vivo in Balb/c mice (△) Bars represent standard errors Reproduced from reference 2 (Shibamoto Y, Ono K, Takahashi M, et al.: An in vitro and in vivo screening system for new hypoxic cell radiosensitizers using EMT6 cells. Jpn J Cancer Res. 1986, 77:1027-33. 10.20772/cancersci1985.77.10_1027) with permission from the publisher

The main reason why single-fraction STI is not considered ideal is tumor hypoxia. Most tumors over a certain size are considered to contain radioresistant hypoxic cells. Due to the existence of hypoxia, the tail of radiation dose-survival curves for in vivo tumor cells becomes parallel to the curves for hypoxic tumor cells. Figure 1 shows the dose-survival curves for aerobic and hypoxic EMT6 cells in vitro and for in vivo EMT6 cells in Balb/c mice [2]. It is clearly shown that the tail of the dose-survival curve for in vivo EMT6 cells is almost parallel to the curve for hypoxic EMT6 cells, and that single high doses of irradiation are not as effective as relatively low doses in terms of the number of in vivo tumor cells killed per Gy. 

Thus, it is clear that tumor hypoxia hampers antitumor efficacy in STI. The question would naturally arise as to how large a tumor needs to be before it becomes hypoxic. It is considered that tumor hypoxia develops at a certain tumor size and increases with increasing tumor size, but not much information is available in the literature about the relationship between hypoxia and tumor size. Most data come from experimental studies with murine tumors. However, classical studies mostly used anesthesia or physical restraint of mice, which is known to artificially increase the hypoxic fraction at the time of irradiation [3]. It is difficult to estimate the true hypoxic fraction from such studies. The author and colleagues have investigated the hypoxic fractions of murine tumors by whole-body irradiation of tumor-bearing mice without physical restraint or anesthesia in a study [4].

**Figure 2:**
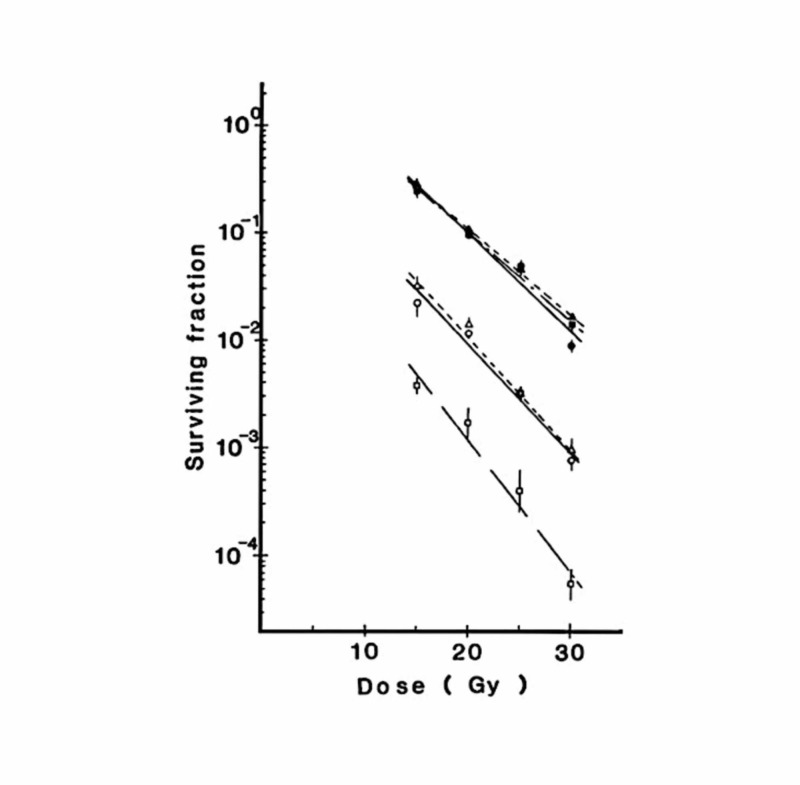
Dose-survival curves for SCCVII tumor cells in C3H/He mice The longest diameters of the tumors were approximately 5 mm (□, ■), 10 mm (○, ●), and 18 mm (△, ▲) Open and closed symbols represent tumors in air-breathing and asphyxiated mice, respectively. Bars represent standard errors Reproduced from reference 4 (Shibamoto Y, Yukawa Y, Tsutsui K, Takahashi M, Abe M: Variation in the hypoxic fraction among mouse tumors of different types, sizes, and sites. Jpn J Cancer Res. 1986; 77:908-15. 10.20772/cancersci1985.77.9_908) with permission from the publisher

Figure 2 shows dose-survival curves for SCCVII cells transplanted subcutaneously in C3H/He mice. By the paired survival curve assay, the hypoxic fraction was estimated to be 0.86% for 5-mm SCCVII tumors, 8.5% for 10-mm tumors, and 8.4% for 18-mm tumors. With an increase in the tumor size, the hypoxic fraction increased but reached a plateau at 10 mm.

The hypoxic fractions were also investigated in three other tumors [4]. The hypoxic fraction of subcutaneously transplanted EMT6 tumors did not reach a plateau at 10 mm but increased up to 16 mm. Intradermal EMT6 tumors had a higher hypoxic fraction than subcutaneous ones. The results of this study indicated that hypoxia developed in tumors with a diameter of 5 mm or smaller.

**Table 1:** Hypoxic fractions of four kinds in tumors of various sizes *95% confidence interval

Tumor	Site	Size, mm	Hypoxic fraction
RIF1	Subcutaneous	10	4.7% (3.3-6.6)*
B16	Subcutaneous	10	4.5% (3.2-6.2)*
SCCVII	Subcutaneous	5	0.86% (0.42-1.7)*
	Subcutaneous	10	8.5% (6.9-11)*
	Subcutaneous	18	8.4% (6.1-12)*
EMT6	Subcutaneous	6	5.9% (3.9-8.8)*
	Subcutaneous	10	14% (9.9-19)*
	Subcutaneous	16	22% (15-33)*
	Intradermal	6	16% (12-21)*

Hypoxia has also been proven to exist in human tumors [5-7], but the lower size limit of tumors with hypoxia has not yet been established. However, considering the existing data and data on murine tumors, it is reasonable to assume that tumors of >1 cm in diameter contain hypoxic cells. With enlargement, the presence of hypoxia would become more definite and the size of the hypoxic fraction would expand. Even if a 1-cm tumor contains hypoxic cells, it can be empirically controlled by single-fraction SRS. So, the authors think that tumors over 2 cm are good candidates for fractionated irradiation.

2. Reoxygenation

**Figure 3:**
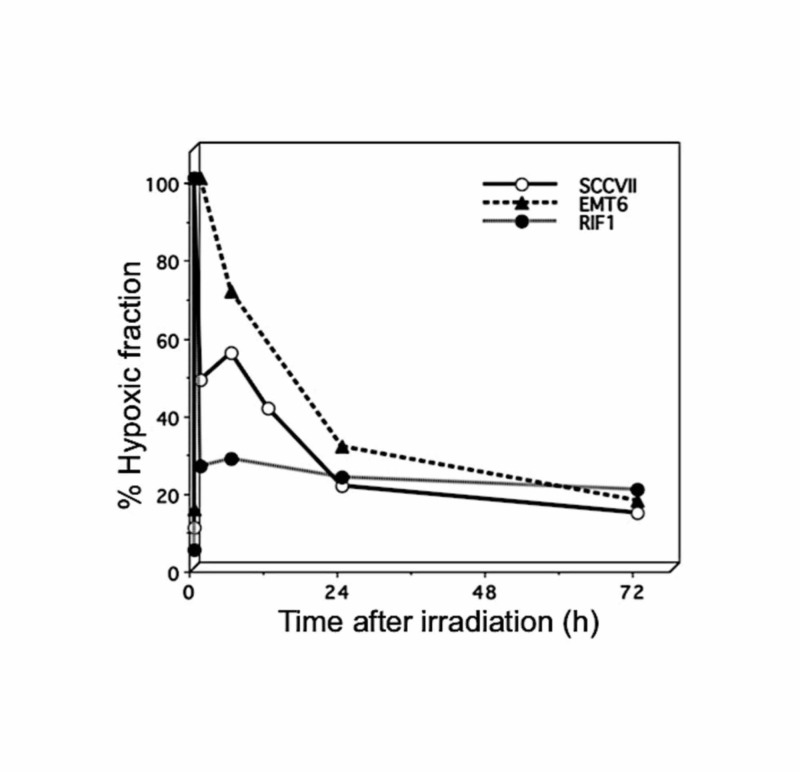
Changes in the hypoxic fraction after single high-dose irradiation in three murine tumors Reproduced from reference 9 (Shibamoto Y, Miyakawa A, Otsuka S, Iwata H.: Radiobiology of hypofractionated stereotactic radiotherapy: what are the optimal fractionation schedules? J Radiat Res. 2016, 57(S1): i76-i82. 10.1093/jrr/rrw015) with permission from the publisher.

The main reason why fractionation is beneficial against large tumors is due to the reoxygenation of surviving hypoxic cells before the next fraction. In rodent tumors, reoxygenation is known to take place relatively quickly after a single high dose of irradiation. Figure 3 shows the changes in hypoxic fractions after a single high-dose (13 or 15 Gy) irradiation in three 1-cm subcutaneously transplanted murine tumors [8].

These data were obtained without using anesthesia or the physical restraint of mice, both of which markedly increase the hypoxic fraction of tumors [3]. In all three tumors, the hypoxic fractions at 24 hours after irradiation were significantly lower than those immediately after irradiation, indicating that reoxygenation was clearly present. In EMT6 tumors, reoxygenation was relatively slow, and it seemed to take longer than 24 hours for the hypoxic fraction to return to the pre-irradiation level. In the other tumors too, the hypoxic fractions tended to decrease further after 24 hours. Taking these results into consideration, we think that a 24-hour interval between fractions of stereotactic radiotherapy (SRT) may not be optimal for every tumor, and longer intervals may allow more reoxygenation to occur in some tumors. Therefore, we recommend using interfraction intervals of 48 or 72 hours in fractionated SRT for lung cancers [8]. 

To utilize the benefit of reoxygenation, more fractions would be beneficial. We previously proposed the concept of the “reoxygenation utilization rate” in fractionated radiotherapy [9]. In single-fraction SRT, the favorable phenomenon of reoxygenation cannot be utilized. In two-fraction treatment, reoxygenation can be utilized after the first fraction but not for the second fraction. So, the reoxygenation utilization rate is 50%. With an increase in the fraction number, this utilization rate goes up to 75% for four-fraction treatment, 83% for six-fraction treatment, and 87.5% for eight-fraction treatment. In 30-fraction treatment, the rate goes up to 96.7%. Hence, the authors think that six- to eight-fraction treatment may be efficient enough to utilize the reoxygenation phenomenon [9]. In future SRT studies, this fact should be taken into account when planning an optimal fractionation schedule.

3. Other factors

Among the “four R's” (repair of DNA damage, redistribution of cells in the cell cycle, repopulation, and reoxygenation of hypoxic tumor areas) considered to play a role in fractionated radiotherapy, reoxygenation is the most important in STI, as stated above. With respect to the other three Rs, repair takes place between the fractions of STI, but it occurs in both tumors and normal tissues. So, the influence of repair is unclear. Repair is also known to take place to some extent during the few minutes between the respective portals of STI when multiple port irradiation methods are employed [10,11]. However, it has been suggested that a decrease of radiobiological effects due to this repair is counterbalanced by rapid reoxygenation that may occur within a few minutes after irradiation [12,13]. Therefore, the influence of this repair during daily treatment is also unclear, and may not be very important.

**Figure 4:**
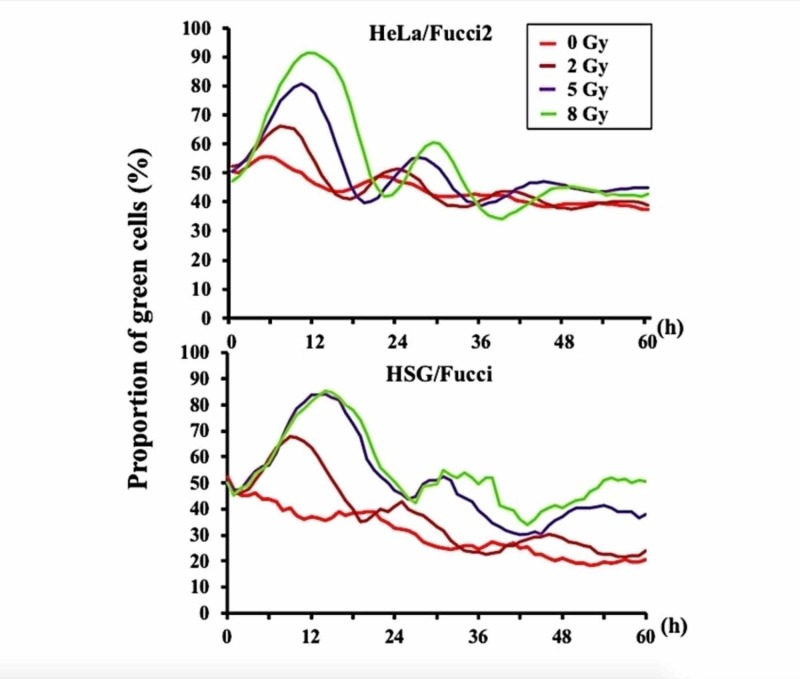
Curves representing the proportion of green S/G2/M phase HeLa/Fucci2 and HSG/Fucci cells after irradiation Modified from the data included in reference 14 (Iwata H, Shuto T, Kamei S, et al.: Effects of proton beams and X rays on the cell cycle of fluorescent ubiquitination-based cell cycle indicator (Fucci)-expressing cells. Int J Radiat Oncol Biol Phys. 2018, 102:e154. 10.1016/j.ijrobp.2018.07.602)

Redistribution of tumor cells in their cell cycle would also take place between fractions of STI. Since late-responding normal tissues are usually not dividing, the phenomenon of redistribution would be beneficial in fractionated SRT. Using the fluorescent ubiquitination-based cell cycle indicator (Fucci) system, we investigated the changes in the cell cycle distribution after irradiation in cultured cells [14]. Figure 4 shows the proportion of S/G2/M phase cells in HeLa/Fucci and HSG/Fucci cells after 0, 2, 5, or 8 Gy of irradiation. The peak of the G2 block was observed at 12 hours or later after 8-Gy irradiation, and the proportion of S/G2/M phase cells returned to the pre-irradiation levels at 18-24 hours [14].

The duration of the G2 block may be longer in in vivo tumor cells, so it may take longer than 24 hours for human tumor cells to complete redistribution after high-dose irradiation [15,16]. Therefore, intervals longer than 24 hours may be beneficial to take advantage of the redistribution in some tumors. 

When the overall treatment time becomes long, the repopulation of tumor cells may hamper the effects of fractionated STI. In rapidly growing tumors, this phenomenon may start during the third week of radiation therapy, although it usually becomes problematic only when the overall treatment time is longer than three weeks [17]. Therefore, it may be advisable to complete STI within three weeks. 

Consideration of optimal fractionation

Based on the above findings, we propose some recommendations pertaining to hypofractionated SRT schedules. The Important points are as follows: 1) to more efficiently utilize the reoxygenation phenomenon, six- to eight-fraction schedules are better than three- or four-fraction schedules; 2) reoxygenation and redistribution may not be completed within 24 hours in some tumors, so interfraction intervals of 48-72 hours may be better than a 24-hour interval; and 3) recent studies have suggested that the linear-quadratic (LQ) model could overestimate the effects of high doses per fraction [11], and this should be taken into account when considering optimal doses using the LQ model. Improving the LQ model and creating a new alternative model were discussed in our previous paper [11], but at present, using the LQ model calculation presuming the overestimation of the effects of high fractional doses may be a reasonable solution [9].

Herein we summarize our observations on optimal fractionation schedules for stage I non-small-cell lung cancer. So far, the standard SBRT dose for lung cancer has been 48 Gy in four fractions in Japan [8]. We now also use 50 Gy in four fractions [18]. Small tumors (<2 cm) may be efficiently treated with this protocol; but for larger tumors, we propose 60 Gy in eight fractions delivered in alternate days. If calculated by LQ formalism with an assumption of an α/β ratio of 10 Gy, 60 Gy in eight fractions corresponds to 48 Gy in four fractions. If an α/β ratio of 3 Gy is assumed, 60 Gy in eight fractions may have weaker effects (against late-responding normal tissues) than 48 Gy in four fractions according to the LQ formalism [11]. However, considering the overestimation of the effects of high fractional doses by the LQ model and reoxygenation between fractions, 60 Gy in eight fractions may be more effective against tumors than 48 Gy in four fractions, while the late normal tissue responses may not be so different [11]. Other conceivable fractionation schedules could constitute 54 Gy in six fractions and 60-66 Gy in 10 fractions for intra- as well as extra-thoracic tumors. However, optimal fractionation schedules may largely depend on the type and histology of tumors and should be further discussed taking tumor and organ characteristics into account. With the refinement of fractionation protocols, we expect that the outcomes of patients treated with SRT will further improve, and SRT will become one of the standard treatments comparable with surgery for many cancers.

## Conclusions

We conclude that fractionated STI is highly beneficial and effective for the treatment of tumors, especially for tumors larger than 2 cm. An SRT schedule that constitutes fractions of six or more may be more efficient than one with fewer fractions to better utilize the phenomenon of reoxygenation of hypoxic tumor cells between fractions. To allow for sufficient reoxygenation and redistribution, interfraction intervals of longer than one day may be efficient. But this should be investigated further in future studies.
